# Early cognitive screening for individuals on the dementia continuum: A novel approach amid current trends

**DOI:** 10.1177/13872877261424289

**Published:** 2026-03-18

**Authors:** Diana M. Urian, Garima Gupta, Micheal E. Battista, Conor J. Wild, Adrian M. Owen

**Affiliations:** 1 Schulich School of Medicine & Dentistry, Department of Neuroscience, 6221 Western University, London, Canada; 2 Department of Psychology, 6221 Western University, London, Canada; 3 Western Institute for Neuroscience, 6221 Western University, London, Canada; 4 Creyos Inc., Toronto, Canada; 5 Department of Physiology and Pharmacology, 6221 Western University, London, Canada

**Keywords:** Alzheimer's disease, cognitive screening tools, dementia, dementia prevention, early detection, mild cognitive impairment

## Abstract

Mild cognitive impairment (MCI) is a critical transitional stage between normal aging and dementia that remains challenging to detect. Many traditional neuropsychological assessments designed for early detection of MCI are time-consuming, require specialized training and/or demonstrate limited validity, making them impractical for widespread use in primary care settings. This article is divided into two phases. The first phase provides a rapid review of the current landscape of cognitive screening tools, while the second presents a novel, fully automated, digital screener based on two tasks from the Creyos cognitive assessment platform. This novel screener has been designed, using machine learning, for rapid administration without clinical supervision. The preliminary findings demonstrate that our two-task screener effectively differentiates between cognitively normal individuals and those at risk of progression along the Alzheimer's disease continuum. Furthermore, validation analyses showed that the screener has high sensitivity and specificity, outperforming many conventional assessments. By offering a brief, accessible, and reliable alternative to standard screening tools, our screener has the potential to enhance early detection efforts and facilitate timely intervention, ultimately improving patient outcomes, reducing the burden on clinicians, and optimizing healthcare resources.

## Introduction

The definition of what constitutes “healthy cognitive aging” has been debated for more than six decades.^1^ Although studies have consistently reported that certain executive functions tend to decline early in the aging process (e.g., fluid intelligence, working memory, attention, and decision-making), there continues to be uncertainty about precisely which benign age-related cognitive impairments reflect alterations that should be considered in histopathological terms.^2–5^ This ambiguity partially stems from the continued use of inconsistent terminology. Over the years, numerous terms have been used to describe the pathological cognitive deterioration that can precede (or occur in the absence of) full-blown dementia, including “aging- associated cognitive decline” (AACD),^6^ “questionable dementia”,^7^ “prodromal Alzheimer's disease”,^8^ “preclinical Alzheimer's disease” (Pre-AD),^9,10^ “mild (neuro) cognitive disorder or decline”,^11^ “age-associated memory impairment” (AAMI),^12^ “cognitive impairment, no dementia” (CIND),^13^ “borderline dementia”,^14^ “very mild dementia”,^15^ “isolated memory impairment”,^16^ and “incipient dementia”.^17^ Despite subtle distinctions among these terms, researchers have increasingly adopted the term mild cognitive impairment (MCI), which is used throughout this article.^18^

During the initial conceptualization of MCI, there was extensive debate about whether it should be recognized as a distinct neurological condition, a symptom of another disorder, or merely an extreme form of normal aging.^19^ MCI is now recognized as a disorder involving cognitive decline that exceeds what is expected given an individual's age and education but does not significantly impair activities of daily living. The condition was conceptualized as a liminal state on the dementia continuum, which includes three stages: Cognitively Normal (CN), MCI, and dementia.^20,21^ While these stages typically progress sequentially, MCI can remain stable without progressing to dementia, and dementia can develop without a preceding MCI phase.^22,23^ Consequently, screening for MCI can be challenging as it requires differentiation from dementia at the more severe end of the continuum, and from “healthy” age-related cognitive decline at the less severe end (see Table 1).

**Table 1.** Core features that distinguish CN, MCI and Dementia.

	Normal cognitive aging (CN)	Mild cognitive impairment (MCI)	Dementia
Objective cognitive decline (i.e., psychometrically measured)	*Absent*	*Present*	*Present*
Subjective cognitive decline (i.e., self-reported)	*Variable*	*Present*	*Variable*
Neuropsychiatric or behavioral changes (e.g., depression and anxiety)	*Absent*	*Absent*	*Present*
Impairment on activities of daily living (e.g., personal hygiene and eating)	*Absent*	*Absent*	*Present*
Neuropathological biomarkers (e.g., amyloid-β deposition and neuronal injury)	*Variable*	*Present*	*Present*

Adapted from Petersen et al. (2014). “*Present”* denotes that the feature is typically observed in the given state or condition, whereas “*Absent”* signifies it is not. “*Variable”* suggests uncertainty regarding the feature's consistency, as individuals may present differently, depending on personal factors and condition severity.

To facilitate accurate detection, multiple organizations have suggested what the clinical criteria for MCI should be, including the Mayo Clinic,^24^ the Expanded/Key Symposium,^25^ the National Institute on Aging-Alzheimer's Association (NIA-AA),^26,27^ the Diagnostic and Statistical Manual of Mental Disorders (DSM-5),^28^ and the International Working Group (IWG).^25^ Each organization differs in terms of suggested classification of biomarkers and MCI subtypes, as well as how much emphasis is placed on activities of daily living, clinical judgment, and neuropsychological performance. While clinicians at specialized neurology and dementia centers may be well equipped to navigate the variability in screening and diagnostic practices for MCI and dementia, the lack of standardized screening protocols poses a challenge in primary care settings, often resulting in missed opportunities for identifying individuals in the early or mild stages of cognitive impairment.^29^

In most primary care settings, physicians rely on subjective complaints (i.e., patient or spouse-reported), and brief symptom observation to determine whether a full neurological assessment is warranted; limited, if any, formal cognitive testing is typically conducted. It has been estimated that 50% of individuals with dementia never received a clinical cognitive evaluation during the early or mild stages of their disease.^30^ This issue is exacerbated by a lack of healthcare policies requiring the use of specific assessment tools or cognitive evaluations by primary care providers.^31^ Indeed, primary care providers often express uncertainty about the most appropriate methods for detecting MCI and therefore tend to rely on direct clinical observation, which, in the absence of sufficient training in MCI and dementia, can lead to conflation of the two conditions.^32–35^ Additionally, primary care providers, who may be managing more than 30 patients per day, lack the resources and training required for comprehensive neuropsychological testing typically conducted in specialized neurology clinics for diagnosis of MCI or dementia.^36–38^ As a result, many patients go untested or are referred to neurological clinics, which may have waiting periods exceeding three years.^39^

Currently, 19% of the global population aged 50 and above is estimated to be living with MCI—a statistic that is projected to increase quickly as the proportion of adults over the age of 65 continues to grow.^40,41^ These numbers confirm that there is an urgent need to transition from a reactive, treatment-based model to a proactive, prevention-focused approach. Such preventative approaches demand optimizing “screening” instruments that can be used routinely in primary care settings to promote early detection of at-risk individuals, estimate disease urgency, implement preventative measures, and reduce errors related to the onset (or otherwise) of full-blown dementia.^42–44^ An effective screening instrument should be able to accurately identify individuals at risk of developing significant health issues prior to them presenting with hallmark symptoms or actively seeking treatment. It would allow clinicians to make referrals for further assessment or intervention in a streamlined manner. To be practically useful, however, a good screener should also be brief, facilitating its use in routine examinations across common settings such as clinics, schools, or workplaces. Several such screeners currently exist and are being used to differentiate between CN, MCI, and dementia with variable success.

In the current paper, the most widely used cognitive approaches for screening dementia symptoms will be reviewed. Following this review, we will then outline an alternative approach that may be more effective at identifying individuals at risk of progressing on the dementia continuum, supported by preliminary evidence from several healthy and clinical populations.

## Rapid review: Methodology

We searched three databases (PubMed, Scopus, and Web of Science) in August 2024. The search criteria were limited to primary research articles, including human qualitative studies and case reports, published in English between 2004 to 2024. The search was focused on articles that included the terms “screeners,” “Alzheimer's disease,” “mild cognitive impairment,” or “age- associated cognitive decline.” We excluded articles that were focused on lifestyle factors, policy, cellular and physiological markers, genetic research, neuroimaging, and any study focused on intervention without investigation of screeners or dementia.

The database search identified 569 relevant citations (see Supplemental Figure 1). Following the exclusion of duplicates, two members of our research team (DMU and GG) independently screened titles and abstracts to exclude irrelevant citations. A total of 258 full-text relevant publications were reviewed and two features were documented for each study: (I) the sample groups tested (i.e., CN, MCI, or dementia), and (II) the cognitive measures administered. The cognitive measures were further categorized into four groups: (I) paper-and-pencil performance-based screeners (*n* = 47), (II) digital measures (*n* = 20), (III) informant-reported measures (*n* = 7), and (IV) full cognitive batteries (*n* = 16).

Paper-and-pencil performance-based screeners included cognitive assessments that were administered and completed in 15 min or less, consistent with the administration time generally considered appropriate for screeners.^45^ We excluded studies focused on assessing cognitive tasks (e.g., clock-drawing tasks, verbal fluency tests, Boston Naming Test, Trail Making Test, Rey-Osterrieth Complex Figure Test) administered in isolation or selectively combined with other tasks from larger cognitive batteries. Digital measures included performance-based screeners and full batteries specifically designed for computerized platforms. Informant-reported measures relied on information collected from caregivers, rather than direct assessments of the individual with experiencing impairments. Finally, full cognitive batteries included comprehensive performance-based measures that are commonly used for diagnostic and prognostic purposes and that require more than 15 min.

## Rapid review: Results

The literature search revealed that the most frequently reported screeners had an average administration time of approximately 8 min (*M* = 7.7 min), and focused on multiple cognitive processes, including memory, language, and visuo-spatial skills (see Table 2). The Mini-Mental State Examination (MMSE) and the Montreal Cognitive Assessment (MoCA) were the most widely used cognitive screeners for individuals on the dementia continuum by some margin. The MMSE appeared in 164 of 258 articles, while the MoCA was featured in 93 of 258 (some studies included both MMSE and MoCA). This result is unsurprising, as a Web of Science search shows that the original MMSE paper^46^ has been cited 74,501 times, and the original MoCA paper^47^ 17,068 times. Google Scholar reports even higher citation counts, with 109,107 and 25,015 citations, respectively.

**Table 2.** Most frequently reported performance-based cognitive screeners.

Screener	Search frequency	Duration	Domains tested
Mini-Mental State Examination (MMSE)^46^	164	5–10 min	Orientation, (working and delayed) memory, language, and visuo-construction abilities
Montreal Cognitive Assessment (MoCA)^47^	93	10 min	Orientation, executive functioning, memory, attention, language, and visuospatial abilities
Addenbrooke's Cognitive Examination (ACE)^48^	21	15 min	Attention, (working and semantic) memory, verbal fluency, language, and visuospatial abilities
Quick mild cognitive impairment (Qmci) screen^49^	10	3–5 min	Orientation, recognition, executive functioning, delayed recall, verbal fluency, and (logical) memory
Test Your Memory (TYM)^50^	10	5–7 min	Orientation, executive functioning, semantic knowledge, calculation, fluency, similarities, visuospatial abilities, and (working and semantic) memory
Six Item Cognitive Impairment Test (6-CIT)^51^	7	2–3 min	Orientation, calculation, attention, (delayed) memory
Brief Cognitive Screening Battery (BCSB)^52^	7	8–10 min	Executive functioning, (incidental, immediate, delayed, and semantic) memory, learning, recognition, and (verbal) fluency
Mini-Cog^53^	7	3 min	Executive functioning and (working) memory
Frontal Assessment Battery (FAB)^8^	6	10 min	Concept formation and abstract reasoning, (verbal) fluency, praxis (motor programming), sensitivity to interference, inhibitory control, and environmental autonomy
Rowlands Universal Dementia Assessment Scale (RUDAS)^54^	6	10 min	Memory, praxis, body orientation, judgement, drawing, and language

We included only those screeners mentioned more than five times in our rapid review. All screeners listed here have been used to assess individuals with normal cognition, mild cognitive impairment and dementia due to Alzheimer's disease, except for the ACE, which was not identified in studies involving MCI. Additionally, most of these screeners were validated in studies on non-Alzheimer's disease dementias, apart from BCSB.

The disproportionate use of the MMSE and MoCA reflects the fact that many studies focus on validating and refining one, or both, of these two measures, rather than exploring alternatives (i.e., a potential anchoring bias within the field). Indeed, our rapid review revealed that most studies using the MMSE and MoCA typically either validated these tools in different languages, assessed modified versions (e.g., Modified MMSE, s-MoCA, Mini-Mental X), or developed new screeners incorporating elements from these measures (e.g., Mini-Clock). Where other screeners were used, sensitivity and specificity assessments frequently included comparisons with the MMSE and/or MoCA, with the assumption that these were reliable reference points.^55^ However, few comprehensive comparative studies have been conducted to support their use in this way and evidence that either the MMSE or MoCA are superior to any other available screener is scarce.

In fact, compelling evidence exists to question the effectiveness and usability of these two popular screening tools. First, scores on the MMSE and MoCA are influenced by cultural and educational factors.^56–59^ For instance, previous studies have reported that in more than 10% of older adults, poor performance on the MMSE can be attributed to factors other than dementia, particularly lower literacy levels.^60^ Although the MoCA scores can be adjusted to account for level of education, the validity of such adjustments has been questioned.^61^ Second, both the MMSE and MoCA are subject to significant ceiling effects and rely on cut-off scores, which may fail to account for individual variability in symptom presentation. This issue was highlighted in a recent study demonstrating that neither the MMSE nor the MoCA were able to predict functional performance in older adults in geriatric inpatient units.^62^ Third, both tests require administration and scoring by a trained professional—a requirement that poses challenges in an overburdened healthcare system.

## Digital measures

The field of dementia screening is increasingly turning to digital and telephone methods of administration to address challenges related to bias, administrative burden, and the accessibility of cognitive assessments (e.g., T-MoCA, ACEmobile, tele-TYM).^63–65^ Digital platforms often provide more detailed data than paper-and- pencil approaches, including response times, attempt counts, and error types. Moreover, administration can be automatically adapted “on the fly” based on a patient's performance, which is difficult to achieve using analogue methods. A recent review reported that 22 of 46 digital cognitive tests for MCI and dementia demonstrated robust diagnostic performance, with sensitivity and specificity exceeding 80%.^66^ Indeed, in many cases, digital cognitive assessments offer comparable or superior accuracy relative to their traditional paper-and-pencil counterparts. For example, the CAMCOG-CAT, which takes 20–30 min, has slightly higher sensitivity and specificity (i.e., 84% and 94%, respectively), than the CAMCOG paper-and-pencil version (i.e., 83% and 82%, respectively).^67^ Furthermore, most digital tools include automated scoring, which reduces biases in scoring and interpretation of the findings.^68,69^ Several recent studies have also demonstrated that older adults with limited technological experience can proficiently use unsupervised devices, producing reliable data comparable to in-clinic assessments.^70–73^

Despite these advantages, digital screeners have yet to be widely adopted in primary care settings, in part because they typically lack established normative databases. Indeed, our rapid review revealed that most screeners, including currently available digital screeners, rely either on small normative databases composed primarily of older adults (> 65 years) or group all individuals above a certain age into a single normative category.^74–76^ Using normative data derived solely from older adults means that these assessments cannot be applied reliably to middle-aged adults, who would benefit the most from early dementia detection and prevention strategies. Meanwhile, the choice of grouping may create artificial boundaries, which reduces statistical power. To provide meaningful information and avoid measurement bias, it is crucial to compare a patient's performance against relevant standardized norms that capture trends observed in the general public. Without a representative normative database, a patient's cognitive deficits may be characterized inaccurately, leading to over- or under-pathologizing and poor prediction of disease progression.

## Novel dementia screening approach: Methodology

Considering the above argumentation, we recently sought to develop a self-administered, digital online cognitive screener to improve the early detection of dementia across diverse populations of aging individuals. The screener uses a machine learning (ML) algorithm trained on data from thousands of individuals who have completed the Creyos cognitive assessment battery. Creyos is an online neuropsychological battery that includes twelve cognitive tasks that have been administered more than 14 million times over the last 15 years. From this archive, a robust normative database has been assembled, comprising approximately 85,000 healthy participants, including 5000 individuals over the age of 65.^36^

Unlike many traditional measures, the Creyos battery assesses cognitive performance on a continuum rather than providing a binary classification (i.e., impaired/unimpaired), and task difficulty is adapted dynamically based on participant performance to maximize scoring efficiency. These features minimize ceiling effects, reduce participant frustration or fatigue, and provide a more nuanced account of impairment type and severity.^77,78^ Furthermore, Creyos does not require clinical oversight, making it practical and cost-effective for primary care settings. One recent study demonstrated that a Creyos assessment was comparable to in-person measures in detecting cognitive impairments in healthily aging midlife and older adults.^78^ Moreover, a recent comparison of in-home self-assessment versus supervised laboratory testing revealed no significant differences between the two approaches.^36^ Nevertheless, the full (12-item) Creyos assessment takes 30–40 min, which is too long for implementation in primary care settings. Accordingly, we leveraged the Creyos platform's large normative database and internal datasets to develop a ML model capable of distinguishing CN individuals from those with age-related cognitive decline, suspected memory impairments, MCI or dementia.

The ML algorithm was optimized and trained using data from healthy individuals in the Creyos normative database (*n* = 2859) and patients from neurology clinics specializing in dementia and age-related cognitive decline (*n* = 1856). This was done to ensure accuracy and generalizability. The classifier architecture comprised two stages: (1) a data preprocessing stage, which involved mean-centering features and applying a power transformation to approximate a Gaussian distribution, and (2) a linear Support Vector Classifier (SVC). This architecture was optimized using nested, repeated stratified K-fold cross-validation, ensuring that all model parameters, including preprocessing parameters, were estimated from the inner training data. In the inner cross-validation loops, a grid search was used to identify the optimal combination of cognitive tasks, data features, and classifier hyperparameters (*C* and *γ*) across all possible configurations. This enabled the identification of a combination that could most effectively distinguish cognitively impaired individuals from healthy controls. Once optimized, the outer loops were used to assess the final model performance, ensuring its ability to generalize to new data. To address class imbalance and prevent bias from the larger healthy dataset, random under-sampling was applied.^77^

Ultimately, the algorithm identified two key tasks, Number Ladder and Feature Match, along with two performance markers from each task (i.e., the final task score and a reaction time–derived feature) as the most effective for distinguishing cognitively impaired from unimpaired individuals (see Table 3). Previous studies have consistently reported deficits in visuospatial working memory among individuals with MCI and dementia of Alzheimer's disease (AD).^79–82^ Likewise, impairments in selective, sustained, and divided attention have been commonly observed among individuals with dementia due to AD.^83–87^

**Table 3.** Cognitive tasks comprising the two-task screener.

Task	Cognitive process	Description	Time required
Number Ladder	Visuospatial working memory	Boxes with numbers appear at different screen locations. The participant must try to remember which number corresponds to each box. After the numbers disappear, they click the boxes in numerical order. Task difficulty adjusts based on performance. The final score is the average number of boxes correctly recalled.	No time limit. The task ends when a participant makes three errors.
Feature Match	Attention	Two boxes appear on the screen, each containing an array of abstract shapes. The participant must determine whether the boxes are identical or different by clicking on corresponding buttons (i.e., match or mismatch). Task difficulty adjusts based on performance. The final score is the sum of the difficulty levels of correct responses minus the sum of the difficulty levels of incorrect responses.	1 min and 30 s

## Novel dementia screening approach: Preliminary results

To validate the screener, we applied the pre-trained model to two published datasets comprised of Creyos cognitive test data from large samples of the general population.^88,89^ Data from a total of 9350 individuals (5709 females and 3641 males), ranging in age from 21 to 97 years old (*Q1* = 32, *Median *= 45, *Q3* = 57), were processed by the screener algorithm. This produced a binary outcome (i.e., positive or negative screener outcome) for each cognitive task. Positive screener outcomes (SCR+) flagged individuals at risk of cognitive impairment, whereas negative screener outcomes (SCR-) indicated that no signs of cognitive impairment were detected. The percentage of cases flagged SCR + from the two-task Creyos screener. Specifically, 2.60% of individuals aged 20–30 years (*n* *=* 1997), 4.30% of individuals aged 30–40 years (*n* = 1886), 6.70% of individuals aged 40–50 years (*n* *=* 1795), 12.30% of individuals aged 50–60 years (*n* = 1929), 20.50% of individuals aged 60–70 years (*n* *=* 1353), and 38.50% of individuals aged 70 + years (*n* *=* 410) were flagged as SCR+.

**Figure 1.**
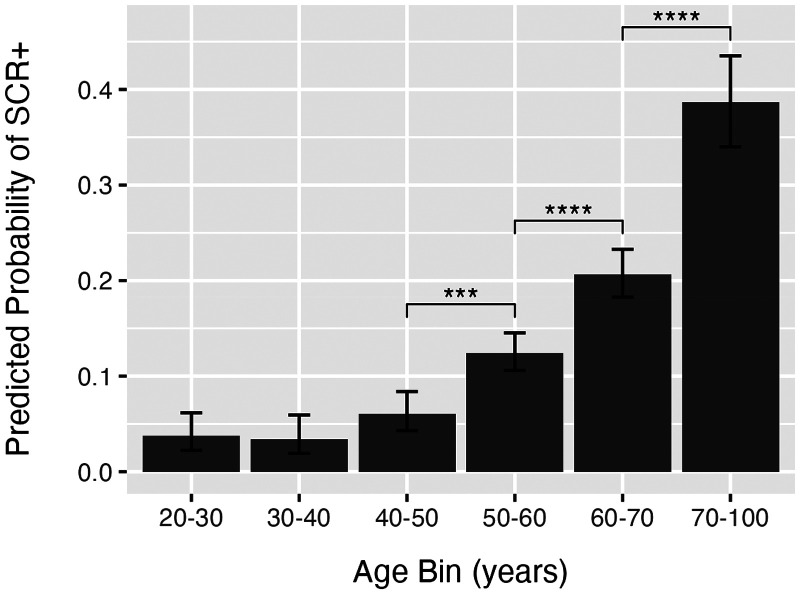
Predicted probability of a positive screener result (SCR+). The predicted probability of a positive screener result (SCR+) from a sample of 9350 individuals, ranging in age from 21 to 97 years. Error bars show 95% confidence intervals of the predicted probability and asterisks indicate significant (*p* < 0.001) post-hoc comparisons between age groups.

The screener outcomes were then analyzed using logistic regression to predict the screener outcome from four factors and their interactions: (I) age bin (20–30, 30–40, 40–50, 50–60, 60–70, 70 + years), (II) sex (male or female), (III) education level (some post-secondary education, or no post-secondary education), and (IV) study.^88,89^ An *ANOVA*-style analysis of these factors was performed using likelihood ratio tests. The analysis revealed a highly significant effect of age (*LR*(5) = 652.50, *p* < 0.001). Post-hoc comparisons of the estimated marginal means revealed that the probability of obtaining a positive result significantly increased for ages over fifty years (see Figure 1). Other factors, including gender, education, and study, did not reveal significant effects, or interactions.

Next, to confirm that the screener was sensitive to cognitive impairment and dementia, we tested the ML model on a dataset of 14 patients that were diagnosed with dementia due to AD by a qualified neurologist using the standard clinical assessments and neuropsychological evaluation procedures. These clinically diagnosed patients then enrolled into a research study (in preparation) that required them to complete the entire 12-item Creyos battery. All 14 patients (100%) were identified as impaired by our screener. Notably, one clinically diagnosed patient in this cohort would not have been classified as impaired based on the MMSE alone (score > 24), highlighting our screener's sensitivity. In a case-matched sample of 14 control subjects, randomly drawn from a normative database of 9350 individuals and matched for age, gender, and level of education, only 2 out of 14 (i.e., 14%) had a SCR+. This outcome corresponds to a specificity of 86%.

A remaining question is whether the screener is specific to dementia-related cognitive impairment. In other words, would different health-related disorders associated with cognitive deficits also trigger positive screener outcomes? To address this, we mined the 9350 datasets described above for individuals who had self-reported having pre-existing health conditions, as diagnosed by their healthcare provider,^89^ or who were considered at risk for generalized anxiety disorder or depression according to screening questionnaires (i.e., the Generalized Anxiety Disorder 2-item, GAD-2, and the Patient Health Questionnaire-2, PHQ-2, respectively).

**Figure 2.**
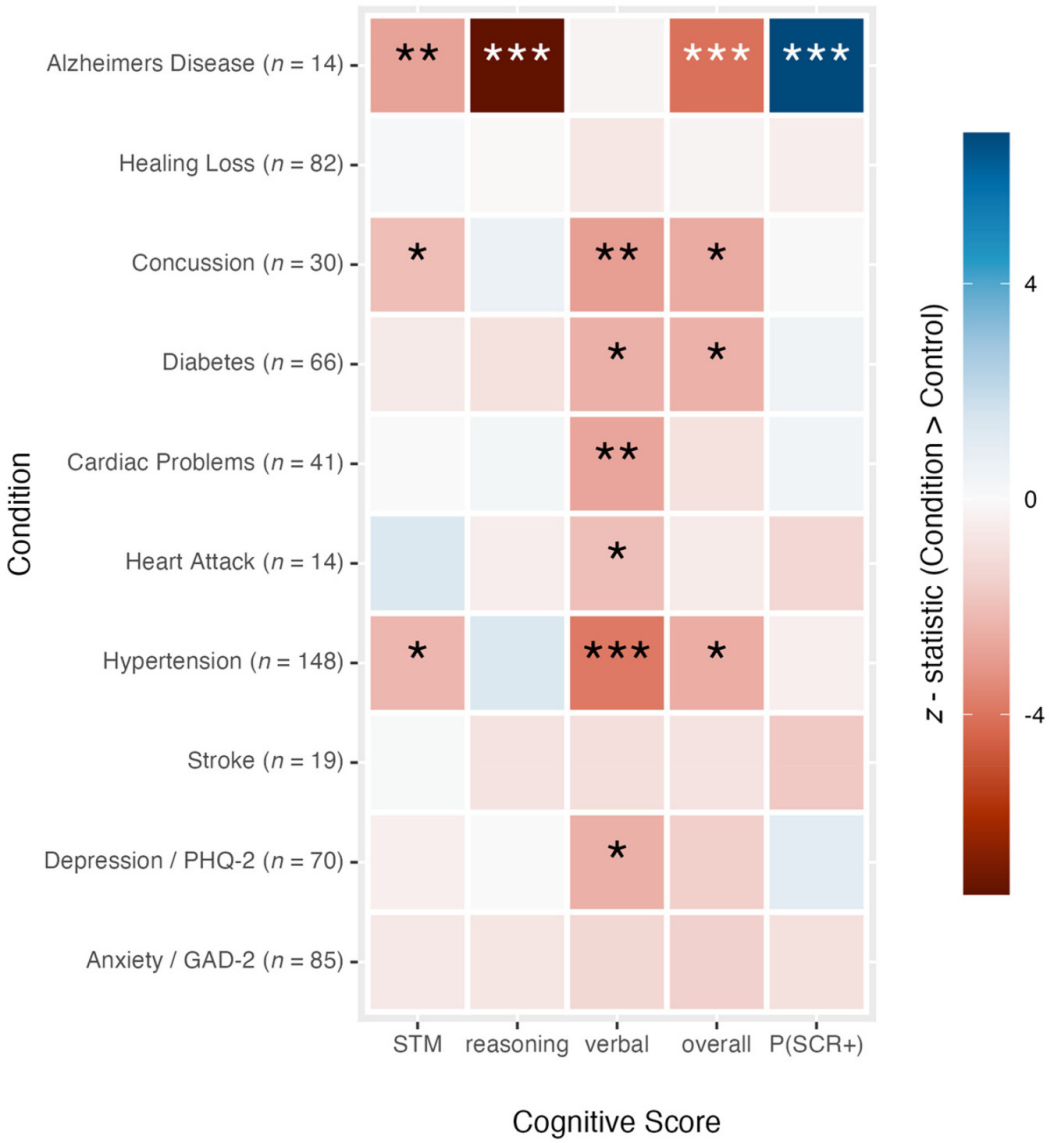
Cognitive outcomes and screener flags for various conditions. A graphical summary of 50 statistical tests comparing groups of individuals with pre-existing health conditions against age, gender, and education matched controls on four measures of cognition and on the probability of being flagged by our 2-task screener (SCR+). For the four cognitive scores—short-term memory (STM), reasoning, verbal, and overall scores—linear regression outcome models were used to compare average scores between groups, whereas the binary screener outcome was compared between groups using a logistic regression outcome model. G-computation was used to estimate the effects and compute *z*-statistics. A negative statistic (warm, red hue) indicates that the condition group had a lower cognitive score (or probability of a SCR + result) than their matched controls, whereas a positive statistic (cool, blue hue) indicates the opposite relationship. Asterisks indicate significant effects uncorrected for multiple comparisons (*p < 0.05, **p < 0.01, ***p < 0.001). When these results are corrected for multiple comparisons using the false discovery rate method, the hypertension, concussion, and cardiac groups remain cognitively impaired on at least one measure of performance. The groups with diabetes, heart attack, and depression missed the significance cut off, while the AD group remained significantly impaired on three of the four cognitive domains, in addition to the screener score. (Color figure available online).

For each disorder with more than 10 individuals (i.e., a condition group), we constructed a sample of healthy control subjects matched on age, sex, and level of education. We compared the groups and the corresponding controls on four composite measures of cognitive performance and on the probability of producing a positive screener result (see Figure 2). Since each Creyos task evaluates multiple cognitive domains simultaneously, and different disorders are associated with distinct impairment profiles, we assessed cognitive performance based on overall domain scores rather than comparing all 12 Creyos tasks individually. Domain scores were calculated using factor loadings from previous research.^77^ This process involved standardizing test scores from the 12 Creyos tasks, applying a power transformation (i.e., Yoe-Johnson), and multiplying the standardized scores by the pseudo-inverse of the Varimax-rotated factor loading matrix. The resulting weighted scores were then summed across tasks to generate an overall score for each cognitive domain.

Several conditions (e.g., hypertension, concussion, and cardiac problems) were associated with statistically significant cognitive impairments, as reflected by lower scores, on average, compared to matched controls in certain domains, indicating a negative difference (i.e., warm-colored cells, red hues in Figure 2). However, despite the impairments, these conditions but were not associated with an increased probability of positive screener results (SCR+). That is, groups with these conditions had lower cognitive scores than controls on some domains but did not produce significantly more positive screener results. In contrast, the 14 AD patients (see Figure 2, top row) also had statistically lower cognitive scores than their control counterparts and were significantly more likely to be flagged by the screener (i.e., SCR+; blue cell). Indeed, every single member of that group was flagged by the screener, as detailed above.

## Discussion

In the present study, we reviewed the current landscape of neuropsychological assessments available for evaluating cognitive impairment in older populations, along with the tools designed for predicting the transition from MCI to dementia. Our findings highlighted that most existing approaches are time-consuming, resource-intensive, and require specialized training for administration and interpretation. Additionally, the MMSE and MoCA remain overly relied upon, despite compelling reasons to adopt alternative assessments. We developed a novel, fully automated screener based on two tasks from the Creyos cognitive assessment platform to address these limitations. Our results demonstrated that this screener has the potential to detect early dementia-related cognitive impairment with high sensitivity and specificity.

Current guidelines recommend early detection of MCI in non-specialist settings, followed by a subsequent multidimensional diagnostic evaluation in secondary care by a specialist—a process known as the “two-step diagnostic strategy”.^29^ However, the feasibility of effectively implementing this strategy remains challenging, with some experts arguing that it may be “inadequate and counterproductive” in diagnosing MCI.^90^ Several concerns have been raised, including the fact that MCI symptoms are more subtle and difficult to detect than those of dementia, particularly by non-specialists and family members.^90^ Given that many early MCI symptoms are non-cognitive, another consideration is that cognitive screening tests are generally less effective at identifying MCI compared to dementia.^90^ Furthermore, the unclear pathological significance of MCI and its potential reversibility can sometimes lead non-specialists to delay or forgo referral to specialists, hindering timely intervention.^90^

Our screening tool aims to enhance the two-step diagnostic strategy by addressing several of these challenges. A key advantage of our screener is its strong predictive validity, with statistical sensitivity and specificity comparable to, or exceeding, many currently available tools. Previous longitudinal studies have assessed the accuracy of the MMSE in identifying individuals with baseline MCI who later progress to AD, vascular dementia, or other forms of dementia. A review of 11 such studies reported substantial variability in MMSE's sensitivity (ranging from 27% to 89%) and specificity (ranging from 32% to 90%) for AD.^91^ Depending on the cut-off scores used, similar variability in sensitivity and specificity has been reported for the MoCA.^53,92,93^ Notably, our screening tool demonstrates a sensitivity of 100% and a specificity of 86% for AD, positioning it at the higher end of these reported ranges. These results demonstrate that our screener is sensitive to dementia and specific enough that healthy controls produce a positive result that aligns with the expected incidence of MCI in the general population, which is estimated to be over 20%.^40^

The higher sensitivity relative to specificity is both expected and desirable in a screening tool, as it addresses one of the primary concerns associated with the two-step diagnostic strategy. While diagnostic tests should prioritize specificity to ensure accurate case identification, a screening tool benefits from higher sensitivity to detect potential cases early.^94^ Several recent studies have suggested that lifestyle modifications,^95,96^ certain types of cognitive training,^97^ and pharmacological interventions^98^ are more likely to be effective when implemented in the earliest stages of cognitive decline. By offering a rapid and reliable method to distinguish normal age-related cognitive decline from pathological impairments, our tool has the potential to facilitate earlier referrals to neurologists and memory clinics.

Beyond predictive validity, our screener demonstrates promising efficiency. Traditional neuropsychological assessments typically require extensive time (i.e., several hours) and expertise, often limiting their accessibility in primary care and general clinical practice. In contrast, our screener can be administered in approximately six minutes and does not require specialized training, making it feasible for widespread implementation in routine medical check-ups. The simplicity and automation of our screener also prevent overburdening specialists and reduce the potential for human error and variability in scoring, increasing the reliability of results across diverse clinical settings. Another key strength of our screener is the incorporation of machine learning, which improves its ability to identify patterns and predict outcomes.^99–102^ Given the growing burden of dementia and the critical need for early detection, integrating such a tool into standard clinical practice could significantly enhance the identification of individuals at risk.

Despite its advantages, several limitations must be acknowledged. Although our study demonstrates promising initial performance metrics, further validation in diverse populations and real-world clinical settings is essential to confirm the generalizability of our findings. Notably, the sample size for the AD cohort was small (*n* = 14), making the reported sensitivity and specificity of the screener preliminary. In addition, while the screener effectively detects cognitive impairment, it is not intended to replace comprehensive neuropsychological evaluations or differentiate between stages of disease severity along the dementia continuum. Instead, it may serve as an accessible first-line tool that informs subsequent testing and intervention. In a clinical context, a positive result from the two-task screener (i.e., “potentially impaired”) could indicate the need for additional neuropsychological testing to characterize the severity and nature of cognitive deficits. Conversely, a negative result from the screener may help prevent overtreatment and reduce the strain on an already burdened healthcare system, while still allowing for long-term monitoring of patients with subjective complaints to ensure they maintain their low-risk profile.

Furthermore, we acknowledge the ongoing debate over allocating limited healthcare resources to introduce and validate dementia screening programs. Some have argued that constant testing induces unnecessary anxiety in older adults, and available resources should be directed toward promoting life-course risk reduction and providing community support.^103^ As such, the ethical considerations regarding who will be impacted by screening and the extent of that impact—viewed from the perspectives of various stakeholders—should always be carefully considered before any widespread adoption of such programs.

Future research should prioritize longitudinal studies to evaluate the long-term predictive value of this screener across diverse populations, with a particular focus on individuals with MCI. To this end, we are currently initiating a large-scale study to validate the two-task screener by tracking the cognitive trajectories of cognitively normal adults, individuals with subjective cognitive decline, MCI, and dementia over 12 months. Inclusion of CN individuals and those with subjective cognitive decline will inform the predictive accuracy of the screener, whereas the MCI and dementia cohorts will allow us to evaluate its sensitivity. Overall, the full analyses will enable a more comprehensive assessment of predictive value, sensitivity across the cognitive continuum, and performance in relation to cognitive reserve, frailty, activities of daily living, and overall physical and mental health.

In addition to longitudinal studies, researchers should also investigate the potential integration of this screener with emerging biomarkers, such as blood-based or digital health indicators, to improve early detection accuracy. Several studies have already begun exploring such integrations. For instance, researchers have been examining serum biomarkers due dementia due to AD, including the Aβ_42/40_ ratio and phosphorylated tau181 concentration, in relation to longitudinal cognitive decline across various cognitive tests.^104^ Other studies have leveraged multimodal learning approaches, such as integrating virtual reality kiosk-derived data with MRI biomarkers, to enhance early detection of MCI.^105^ A third future direction involves assessing the feasibility of integrating the screener into an electronic health record system to optimize routine clinical care. Integration of the two-task screener in primary care settings has the potential to simplify initial screening and longitudinal monitoring, thus improving patient outcomes and clinical workflow efficiency.

## Conclusion

Based on the insights from a rapid literature review, we have developed a two-task screener for the early detection of cognitive impairment, addressing key limitations of existing screening methods for individuals on the dementia continuum. The combination of high accuracy, efficiency, and accessibility makes our two-task screener ideal for use in primary care settings. By overcoming the constraints of traditional neuropsychological assessments, this approach has the potential to facilitate earlier detection, improve patient care through timely interventions, and support broader public health efforts to mitigate the impact of dementia on aging populations.

## Supplemental Material

sj-docx-1-alz-10.1177_13872877261424289 - Supplemental material for Early cognitive screening for individuals on the dementia continuum: A novel approach amid current trends Supplemental material, sj-docx-1-alz-10.1177_13872877261424289 for Early cognitive screening for individuals on the dementia continuum: A novel approach amid current trends by Diana M. Urian, Garima Gupta, Micheal E. Battista, Conor J. Wild and Adrian M. Owen in Journal of Alzheimer's Disease
