# Supplementary material for: Early cognitive screening for individuals on the dementia continuum: A novel approach amid current trends
Source: J Alzheimers Dis. 2026 Mar 18;110(4):1547–59. doi: 10.1177/13872877261424289 (PMC13058158; doi:10.1177/13872877261424289)
Supplement: sj-docx-1-alz-10.1177_13872877261424289 - Supplemental material for Early cognitive screening for individuals on the dementia continuum: A novel approach amid current trends [file sj-docx-1-alz-10.1177_13872877261424289.docx]

**Supplementary Material**

**Early cognitive screening for individuals on the dementia continuum: A novel approach amid current trends**

**Supplemental Figure 1.** Literature search strategy review

**Identification of studies via databases**

Records removed *before screening*:

Duplicate records removed (*n* = 266)

Records identified from:

PubMed (*n* = 157)

Scopus (*n* = 223)

Web of Science (*n* = 189)

**Identification**

ABS("cognitive screen*" OR "neurocognitive screen*" OR "neuropsychological screen*") AND ABS("Alzheimer's disease" OR "mild cognitive impairment" OR "age-associated cognitive decline") AND NOT ABS("review*" OR "recruitment" OR "pharmacolog*" OR "polic*" OR "regulat*" OR "internal medicine" OR "surgery" OR "subcortical dementi*" OR "histopatholog*" OR "sleep" OR "exercise" OR "smoking" OR "diet" OR "molecular" OR "protein*" OR "antibod*" OR "glia*" OR "gene*" OR "cell*" OR "connectivity*" OR "imaging" OR "stimulation" OR "network*" OR "drug*" OR "medication*" OR "medicine" OR "treatment" OR "therapy" OR "intervention" OR "Parkinson's" OR "vascular" OR "fronto-temporal" OR "depression" OR "drive" OR "delirium" OR "oral" OR "rehabilitation" OR "brain injury" OR "HIV" OR "hypertension" OR "cardio*" OR "cardiac" OR "stroke" OR "tau" OR "amyloid" OR "plaques" OR "beta")

**Inclusion**

**Filtering**

Records excluded (*n* = 45)

Records screened from titles and abstracts

(*n* = 303)

Studies included in full-text review (*n* = 258)
